# Trypanosomiasis Relapse after Melarsoprol Therapy, Democratic Republic of Congo, 1982–2001

**DOI:** 10.3201/eid1106.050036

**Published:** 2005-06

**Authors:** Jacques Pépin, Bokelo Mpia

**Affiliations:** * Centre for International Health and Department of Microbiology and Infectious Diseases, University of Sherbrooke, Sherbrooke, Quebec, Canada;; † Nioki Hospital, Nioki, Democratic Republic of Congo

**Keywords:** Trypanosoma brucei gambiense, melarsoprol, Democratic Republic of Congo, African trypanosomiasis

## Abstract

Recently, a high proportion of patients with late-stage *Trypanosoma brucei gambiense* trypanosomiasis, who had been treated with melarsoprol in some disease-endemic areas, subsequently relapsed. To determine whether the frequency of postmelarsoprol relapses increased over time, we reviewed data from 2,221 trypanosomiasis patients treated with melarsoprol during this period in Nioki, Democratic Republic of Congo, from 1982 to 2001. The frequency of relapses was 5.6%(31/553), 6.8%(35/512), 4.5%(18/398), 11.4%(34/299), and 5.0%(17/343) for those treated from 1982 to 1985, 1986 to 1989, 1990 to 1993, 1994 to 1997, and 1998 to 2001, respectively. The higher frequency of relapses in 1994 to 1997 was associated with an incremental dosage regimen of melarsoprol. In multivariate analysis, after adjustment for treatment regimen, sex, residence, and trypanosomes in cerebrospinal fluid, postmelarsoprol relapses did not increase in Nioki, perhaps because 1) little drug pressure exists; 2) subtherapeutic doses have rarely been administered; 3) little potential exists for the preferential transmission of melarsoprol-resistant strains.

Recent reports suggest that the frequency of relapses after melarsoprol treatment of late-stage *Trypanosoma brucei gambiese* trypanosomiasis may be increasing, at least in 3 foci where the frequency of postmelarsoprol relapses is higher than the 5%–8% generally seen elsewhere (1). In Ibbe, southern Sudan, 18%–20% of patients treated with melarsoprol eventually relapsed (2,3), as did 27% of similar patients in the Arua focus of northwestern Uganda (4). In M'banza Congo, northern Angola, 25% of patients treated with melarsoprol had cerebrospinal fluid (CSF) positive for parasites during treatment or relapsed within 1 month (5). This development could have serious implications for countries highly endemic for *T.b. gambiense* trypanosomiasis (6,7). Eflornithine is the only alternative drug to melarsoprol for patients in late-stage trypanosomiasis, generally defined by either the presence of trypanosomes in the CSF, a CSF leukocyte count higher than 5/mm^3^, or both, but this drug needs to be administered intravenously every 6 hours for 14 days, not an easy task for rural hospitals with limited human and financial resources.

An important question is whether rates of postmelarsoprol relapses are indeed rising in these foci or merely reflect a long-standing decrease in susceptibility to melarsoprol of local strains, which became more obvious when the incidence increased or when data collection and analysis improved. Anecdotal reports have indicated that the frequency of failures was 40% among patients treated with melarsoprol in the early 1970s in the Kimpangu hospital of Zaire (8). This hospital is located at the Angolan border, and many of its patients came from northern Angola. In Uganda and Sudan, no reports about rates of treatment failures have been published before these recent reports. In Nioki, Democratic Republic of Congo (DRC), data have been collected since 1982 on all patients in whom Gambian trypanosomiasis was diagnosed. We thus reviewed our database to determine whether secular changes had occurred in the frequency of postmelarsoprol relapses.

## Methods

Nioki hospital, the only hospital of the district (population ≈110,000), is located in the Bandundu Province of DRC, at the confluence of the Mfimi and Molibampe Rivers. Several trypanosomiasis foci are located on the Mfimi River (inhabited by the Basakata) while others lie on the Molibampe River (populated by the Bampe) or on the road going north from Nioki (populated by the Baboma and the Basengele). These 3 areas, as well as Nioki town (population ≈35,000), represent natural subdivisions of the Nioki District. Patients from Nioki town can acquire their infection either in Nioki itself, where there are infective tsetse flies, or when traveling in rural parts of the district.

All patients with *T.b. gambiense* trypanosomiasis treated in Nioki hospital from January 1, 1982, to December 31, 2001, were included in this observational study; follow-up data were accumulated until December 31, 2003. Most cases were diagnosed at Nioki hospital, while a minority were referred by case-finding mobile teams or rural health centers. Case-finding teams were active in the 1980s, collapsed around 1991 (9), and were reorganized on a more modest scale later on. The vast majority of patients lived within Nioki District, but case-patients from disease-endemic villages outside the district have also been included. The routine work-up of suspected case-patients included a lymph node aspirate when cervical lymphadenopathy was present and a lumbar puncture for CSF examination. Examinations of blood were performed only if other assays failed to show trypanosomes. Patients were considered to be in early stage if the CSF leukocyte count was 1–5/mm^3^, or in late stage when CSF leukocyte count was >5/mm^3^, and treatment was selected accordingly.

Melarsoprol was used throughout the study period for most adult patients with late-stage trypanosomiasis; a small number were treated with eflornithine during clinical trials of this drug. Until mid-1985, late-stage disease in children was treated with trimelarsan. Later, trimelarsan was no longer available, and melarsoprol was used for children as well. From 1984 to 1988, half of melarsoprol-treated patients also received prednisolone during a randomized controlled trial (10); thereafter, prednisolone was given to all melarsoprol-treated patients, except for brief periods of drug shortage. Details of treatment schemes are available in other publications (10–13), in which various parts of the cohort of patients reviewed here were reported. Most patients were given 2 or 3 series (separated by 1-week drug-free intervals) of 3 or 4 daily injections of intravenous melarsoprol at the full dosage (3.6 mg/kg, for a maximum of 180 mg), depending on the CSF leukocyte count. During shortages of pentamidine and suramin, melarsoprol (1 series of 3 injections) was sometimes used in the treatment of early-stage patients. From April 1996 to December 2001, a trial of 3 regimens of melarsoprol took place (14), and consenting patients were randomized to receive either the conventional dosage (3 series of 3 injections of 3.6 mg/kg), a new regimen of 10 consecutive daily injections of 2.16 mg/kg (15), or a regimen of 3 series of 3 injections in which an incremental dosage was used (1.8, 2.16, 2.52; then 2.52, 2.88, 3.24; then 3.6 mg/kg for the last 3 injections). Enrollment in the latter arm of the study was terminated prematurely in December 1998 when a high relapse rate became apparent (14).

After treatment, patients were followed up with lumbar punctures every 6 months for 2 years. Most of the follow-up lumbar punctures were performed at Nioki hospital; in the earlier years, some of these were performed by mobile teams during visits to disease-endemic villages. The patients were asked to come back sooner if they experienced symptoms compatible with a relapse (somnolence, constant headaches). The decision to administer a second treatment (and thus to consider this case as a relapse) was left to the discretion of the attending physician, but most fulfilled one of the following criteria: 1) trypanosomes found in the CSF (or rarely in the blood or lymph node aspirate); 2) CSF leukocyte count ≥50/mm^3^ and higher than the previous determination; or 3) CSF leukocyte count of 20 to 49/mm^3^, higher than the previous determination, with the presence of symptoms compatible with a relapse. When in doubt, the lumbar puncture was repeated 1–2 months later. In practice, distinguishing a genuine relapse from a reinfection is not possible, and we will use "relapse" to designate both. The primary analysis of risk of relapsing considered all relapses, regardless of the interval since melarsoprol treatment; in a secondary analysis, only relapses happening within 2 years of treatment were considered.

For each case, we collected data on age, sex, village of residence, date of diagnosis, mode of diagnosis (lymph node aspirate, blood examinations [wet smear, thick smear, hematocrit centrifugation technique, or any combination thereof], CSF examination), pretreatment CSF leukocyte count, treatment given, encephalopathy or death during treatment, and whether a diagnosis of relapse was made during posttreatment follow-up. Few data were missing with the exception of the village of residence (missing for 61 of 164 patients in 1982) and the precise age (unknown for 1 child and 152 adults). The database did not include information on each follow-up lumbar puncture, but only on those which led to a diagnosis of relapse. Thus, to estimate the frequency of relapses, we used as the denominators all patients who survived treatment.

Data were entered and verified on EpiInfo 6.04, and analyzed with Stata 8.0 (Stata Corporation, College Station, TX, USA). Proportions were compared with the χ^2^ test. CSF leukocyte counts that had a non-normal distribution were compared with rank sum tests. Multivariate analysis was performed by logistic regression; variables that enhanced the fit of the model at the 0.05 level by using the likelihood ratio test were retained.

## Results

Table 1 summarizes sociodemographic, clinical, and biologic characteristics of all patients during each 4-year period from 1982 until 2001. Several of the changes reflect the more systematic case-finding by mobile teams in the earlier half of this period: over time, the proportion of patients with a normal CSF leukocyte count decreased considerably, as did the proportion of patients with a positive lymph node aspirate, while the proportion of patients with CSF trypanosomes increased. The higher proportion of cases in women in the earlier years and subsequent decrease can be explained by this factor (women participate more in case-finding surveys) as well as by a shift in the distribution of cases, with a progressively higher proportion of cases in inhabitants of Nioki town (due to more men migrating out of villages, rural communities have more women then men, but Nioki town does not). Changes in the proportion of patients with trypanosomes in the blood were less striking; because examinations of blood were not conducted for all patients, these variations might reflect changes in the propensity of clinicians to order such assays.

**Table 1:** Demographic and clinical characteristics of all patients with *Trypanosoma brucei gambiense* trypanosomiasis treated in Nioki hospital, 1982–2001*

Characteristic	1982–1985 (%) (n = 1,074)	1986–1989 (%) (n = 889)	1990–1993 (%) (n = 535)	1994–1997 (%) (n = 447)	1998–2001 (%) (n = 374)	p value
Sex						<0.001
Females	669 (62)	472 (53)	283 (53)	217 (49)	161 (43)	
Males	405	417	252	230	213	
Age (y)						<0.001
≤14	239 (25)	178 (20)	91 (18)	69 (15)	63 (17)	
15–49	569 (60)	600 (68)	353 (69)	330 (74)	267 (71)	
≥50	146 (15)	105 (12)	66 (13%)	47 (11)	44 (12)	
Area of residence						<0.001
Nioki town	152 (15)	212 (24)	150 (28)	105 (23)	114 (30)	
Mfimi River	585 (58)	329 (37)	151 (28)	129 (29)	119 (32)	
Molibampe River	78 (8)	79 (9)	60 (11)	72 (16)	49 (13)	
Baboma/Basengele	137 (14)	146 (16)	58 (11)	62 (14)	43 (11)	
Out of district	61 (6)	123 (14)	116 (22)	79 (18)	49 (13)	
Trypanosomes in lymph node aspirate						<0.001
Yes	624 (58)	415 (47)	180 (34)	160 (36)	130 (35)	
No	450	474	355	287	244	
Trypanosomes in blood						<0.001
Yes	313 (29)	341 (38)	157 (29)	138 (31)	126 (34)	
No	761	548	378	309	248	
Trypanosomes in CSF						<0.001
Yes	135 (13)	278 (31)	246 (46)	239 (53)	187 (50)	
No	939	611	289	208	187	
CSF leukocyte count (per mm^3^)						<0.001
1–5	407 (38)	286 (32)	75 (14)	32 (7)	26 (7)	
6–19	188 (18)	120 (13)	38 (7)	34 (8)	38 (10)	
20–99	189 (18)	144 (16)	122 (23)	118 (26)	109 (29)	
≥100	289 (27)	339 (38)	300 (56)	263 (59)	201 (54)	
Median CSF leukocyte count (per mm^3^)	12	31	120	130	112	<0.001

*CSF, cerebrospinal fluid.

Table 2 shows the same characteristics, but only for patients who were treated with melarsoprol. Similar changes in distribution of cases according to sex and area of residence were seen. Even though 96% of these patients were given melarsoprol because they had a CSF leukocyte >5/mm^3^, a shift occurred over time towards more advanced disease upon diagnosis in recent years, as evidenced by the decrease in the proportion with a positive lymph node aspirate, an increase in the proportion with CSF trypanosomes, and a doubling of the median CSF leukocyte count. Treatment regimens varied considerably over time. The proportion of patients given prednisolone also increased after the randomized controlled trial was conducted (10), and its results generalized; the proportion of patients in whom a melarsoprol-induced encephalopathy developed decreased to 2% in the last 4-year period. The proportion of patients found to relapse, whether all relapses were considered or only those occurring within 2 years of diagnosis, increased significantly during 1994–1997, but decreased to its previous level from 1998 to 2001.

**Table 2:** Demographic and clinical characteristics of patients with Trypanosoma brucei gambiense trypanosomiasis treated with melarsoprol in Nioki hospital, 1982–2001*

	1982–1985 (%) (n = 587)	1986–1989 (%) (n = 543)	1990–1993 (%) (n = 417)	1994–1997 (%) (n = 320)	1998-2001 (%) (n = 354)	p value
Sex						<0.001
Females	368 (63)	269 (50)	214 (51)	143 (45)	151 (43)	
Males	219	274	203	177	203	
Age						0.003
≤14	84 (16)	115 (21)	74 (19)	62 (19)	62 (18)	
15–49	341 (66)	354 (66)	272 (69)	229 (72)	254 (72)	
≥50	95 (18)	71 (13)	48 (12)	28 (9)	38 (11)	
Area of residence						<0.001
Nioki town	94 (17)	117 (22)	116 (28)	79 (25)	104 (29)	
Mfimi River	304 (54)	201 (37)	112 (27)	88 (28)	113 (32)	
Molibampe River	25 (4)	45 (8)	48 (12)	47 (15)	48 (14)	
Baboma/Basengele	95 (17)	91 (17)	46 (11)	46 (14)	42 (12)	
Out of district	43 (8)	89 (16)	95 (23)	60 (19)	47 (13)	
Trypanosomes in lymph node aspirate						<0.001
Yes	323 (55)	232 (43)	134 (32)	112 (35)	125 (35)	
No	264	311	283	208	229	
Trypanosomes in blood						<0.001
Yes	151 (26)	197 (36)	106 (25)	88 (28)	114 (32)	
No	436	346	311	232	240	
Trypanosomes in CSF						<0.001
Yes	116 (20)	237 (44)	220 (53)	179 (56)	184 (52)	
No	471	306	197	141	170	
CSF leukocyte count (per mm^3^)						<0.001
1–5	22 (4)	23 (4)	4 (6)	16 (5)	9 (3)	
6–19	158 (27)	114 (21)	30 (7)	18 (6)	38 (11)	
20–99	161 (27)	131 (24)	106 (25)	89 (28)	109 (31)	
≥100	246 (42)	275 (51)	257 (62)	197 (62)	198 (56)	
Median CSF leukocyte count (per mm^3^)	65	101	135	140	116	<0.001
Melarsoprol regimens						<0.001
1 series of 3 injections	17 (3)	7 (1)	26 (7)	14 (5)	5 (1)	
2 series of 3 injections	163 (29)	123 (24)	31 (8)	11 (4)	3 (1)	
3 series of 3 injections	152 (27)	353 (69)	341 (86)	192 (64)	184 (54)	
3 series of 4 injections	221 (40)	29 (6)	0	0	0	
3 series of 3, incremental	0	0	0	44 (15)	24 (7)	
10 daily injections	0	0	0	38 (13)	127 (37)	
Prednisolone						
Yes	177 (30)	339 (62)	341 (82)	320 (100)	354 (100)	
No	410	204	76	0	0	
Melarsoprol-induced encephalopathy						0.002
Yes	31 (5.3)	35 (6.4)	9 (2.2)	11 (3.4)	8 (2.3)	
No	556	508	408	309	346	
Death during treatment						0.25
Yes	34 (5.8)	31 (5.7)	19 (4.6)	21 (6.6)	11 (3.1)	
No	553	512	398	299	343	
All relapses after treatment†						0.004
Yes	31 (5.6)	35 (6.8)	18 (4.5)	34 (11.4)	17 (5.0)	
No	522	477	380	265	326	
Relapses within 2 y of treatment†						<0.001
Yes	21 (3.8)	29 (5.7)	15 (3.8)	32 (10.7)	16 (4.7)	
No	532	483	383	267	327	

*CSF: cerebrospinal fluid. †Excluding patients who died during treatment.

The risk factors for postmelarsoprol relapses during this 20-year period are shown in Table 3. Patients who died during treatment were excluded. In univariate analyses, relapses were significantly more common in male patients, in patients with CSF trypanosomes, in patients treated from 1994 to 1997, and in patients treated with the incremental dosage regimen; relapses were much less common in patients from the Baboma/Basengele subdistrict. Table 3 also displays the results of the multivariate analysis. Associations between relapses and male sex, CSF trypanosomes, residence elsewhere than the Baboma/Basengele subdistrict, and treatment with the incremental dosage regimen were little altered by adjustment for confounding factors and remained statistically significant. When results were adjusted for these confounders (especially treatment regimens), diagnosis from 1994 to 1997 was no longer associated with a higher probability of relapse. The univariate association between CSF leukocyte count and relapses was also strongly confounded by treatment regimens. Removing from the analysis the small number of patients with a normal pretreatment CSF leukocyte count had no impact on any of the adjusted odds ratios (data not shown).

**Table 3:** Risk factors for relapses following treatment with melarsoprol (excluding patients who died during treatment)*

	Relapses/total (%)	Odds ratios (95% CI)	Adjusted odds ratios (95% CI)
Sex			
Females	55/1,091 (5.0)	1.00	1.00
Males	80/1,014 (7.9)	1.61 (1.13–2.30)†	1.57 (1.08–2.27)†
Age (y)			
≤14	19/382 (5.0)	1.00	
15–49	97/1,380 (7.0)	1.44 (0.87–2.39)	
≥50	14/257 (5.4)	1.10 (0.54–2.24)	
Year of diagnosis			
1982–1985	31/553 (5.6)	1.00	1.00
1986–1989	35/512 (6.8)	1.24 (0.75–2.04)	0.83 (0.45–1.52)
1990–1993	18/398 (4.5)	0.80 (0.44–1.45)	0.47 (0.23–0.97)†
1994–1997	34/299 (11.4)	2.16 (1.30–3.59)†	0.82 (0.40–1.69)
1998–2001	17/343 (5.0)	0.88 (0.48–1.61)	0.31 (0.13–0.71)†
Area of residence			
Nioki town	40/486 (8.2)	1.00	1.00
Mfimi River	62/783 (7.9)	0.96 (0.63–1.45)	1.04 (0.67–1.61)
Molibampe River	13/197 (6.6)	0.79 (0.41–1.51)	0.76 (0.39–1.48)
Baboma/Basengele	4/302 (1.3)	0.15 (0.05–0.42)‡	0.14 (0.05–0.40)‡
Out of district	16/311 (5.1)	0.60 (0.33–1.10)	0.59 (0.32–1.10)
Trypanosomes in lymph node aspirate			
No	77/1,217 (6.3)	1.00	
Yes	58/888 (6.5)	1.03 (0.73–1.47)	
Trypanosomes in CSF			
No	62/1,233 (5.0)	1.00	1.00
Yes	73/872 (8.4)	1.73 (1.22–2.45)†	1.52 (1.02–2.27)†
CSF leukocyte count (per mm^3^)			
1–5	1/94 (1.1)	0.23 (0.03–1.79)	
6–19	15/341 (4.4)	1.00	
20–99	39/572 (6.8)	1.59 (0.86–2.93)	
≥100	80/1,098 (7.3)	1.71 (0.97–3.01)	
Melarsoprol regimens			
1 series of 3 injections	1/69 (1.4)	0.21 (0.03–1.53)	0.29 (0.04–2.18)
2 series of 3 injections	11/331 (3.3)	0.49 (0.26–0.93)†	0.47 (0.23–0.95)†
3 series of 3 injections	80/1,222 (6.5)	1.00	1.00
3 series of 4 injections	15/250 (6.0)	0.91 (0.52–1.61)	0.62 (0.30–1.26)
3 series of 3, incremental	16/68 (23.5)	4.39 (2.40–8.04)	4.68 (2.26–9.69)
10 daily injections	12/165 (7.3)	1.12 (0.60–2.10)	1.58 (0.74–3.38)
Prednisolone			
No	33/643 (5.1)	1.00	
Yes	102/1,462 (7.0)	1.39 (0.93–2.08)	

*CI, confidence interval; CSF, cerebrospinal fluid. †p<0.05. ‡p<0.001.

## Discussion

In the Nioki focus of DRC, the frequency of failure of melarsoprol therapy among patients with late-stage *T. b. gambiense* trypanosomiasis increased in 1994 to 1997 but returned to its normal level in 1998 to 2001. This was driven essentially by a high risk for relapse among patients participating in a randomized trial who were treated with a regimen of incremental dosage of melarsoprol (14). The risk for relapse decreased as soon as enrollment of patients in this arm of the trial was terminated. This overall stability in the frequency of postmelarsoprol relapses is remarkable considering that over time the pretreatment characteristics of patients changed in ways (a higher proportion of cases among men, more patients with CSF trypanosomes) that should normally have led to an increase in the risk for relapse. These confounding factors and the otherwise stable crude risk for relapse resulted in significantly lower odds of relapse in patients treated in 1990 to 1993 and 1998 to 2001 compared to those treated at the beginning of the study period. The crude risk for relapse among patients treated in Nioki in 1998 to 2001 was similar to that reported among patients treated in Léopoldville (Kinshasa) 50 years ago (16).

We did not have information on the completeness of follow-up for each patient in the cohort. However, previous studies in Nioki in which this was measured have consistently shown that >80% of patients were followed up for ≥2 years after initial treatment (14,17). During the study period, Nioki hospital was the only one in that part of DRC to offer treatment with eflornithine to patients who relapsed after treatment with melarsoprol. It thus seems unlikely that relapses among our patients would have been identified and managed by other healthcare providers. Some relapsing patients might have died at home without a diagnosis being made, but given the slow course of the disease and the short distances to Nioki hospital, this situation probably did not occur very often. Thus, although using as denominators all patients who survived melarsoprol treatment might have somewhat underestimated the true frequency of relapses, we think that this bias was not substantial and probably did not change over time.

At least 3 factors that generally contribute to the emergence of resistance to antimicrobial agents are not found in the context of the treatment of African trypanosomiasis, which probably explains the stable frequency of postmelarsoprol relapses in Nioki over 2 decades. First, little drug pressure exists: melarsoprol is not used to treat any other condition, and supplies are controlled by a national organization, according to the number of cases reported by each health facility. Second, subtherapeutic doses have not been administered on any sizeable scale; on the contrary, most patients have probably been overtreated ever since the drug became available (15,18). Third, little potential exists for the preferential transmission of melarsoprol-resistant strains because patients with relapses rarely harbor trypanosomes in the bloodstream or lymph nodes, only in the CSF. Such patients are likely not very infectious.

As in Uganda and Angola (4,19), we found an association between the presence of CSF trypanosomes and failure of melarsoprol. Given the limited CSF penetration of melarsoprol, modest geographic variations in in vitro susceptibility might have an impact on the frequency of postmelarsoprol relapses. Twenty-four hours after the administration of melarsoprol, plasma levels are in the range of 2-4 μg/mL, while CSF levels are much lower, from 0.02 to 0.07 μg/mL (18,20,21). For 12 isolates of *T.b. gambiense* from northwestern Uganda, the MIC of melarsoprol was higher (0.009–0.072 μg/mL) than for 2 isolates from Côte d'Ivoire (0.001–0.018 μg/mL); the higher MIC of 0.072 μg/mL was superior to levels that can be expected in CSF (22). Fifty percent inhibitory concentrations measured in *T.b. gambiense* isolates from northwestern Uganda were also higher than in isolates from Côte d'Ivoire (23). In such circumstances, CSF concentrations of melarsoprol might be insufficient to eliminate all parasites. So far, no evidence that genetically determined variations in drug pharmacokinetics might explain this heterogeneity in the frequency of postmelarsoprol failures (21,23). Within the Nioki focus, a lower risk for melarsoprol failure in the Baboma/Basengele subdistrict has been consistently noted for more than a decade (11); we speculate that this might reflect a limited exchange of trypanosomes between the various foci, in which case the in vitro susceptibility of the initial strain(s) in a given focus would be maintained over the years.

Finally, our results suggest that variations in the regimens of melarsoprol used in each country may also explain some, but not all, of the high rates of postmelarsoprol failures in specific locations. A regimen of incremental dosage was indeed used in Uganda (4) and in some patients at Kimpangu hospital, Zaire (24). Whether graded dosing was used in Sudan is unclear. In Angola, the same regimen (3 series of 4 injections: 1.2, 2.4, 3.6, 3.6 mg/kg for each series) was used in M'banza Congo, where 25% of patients relapsed (5), and in Dondo, where only 5%–6% of patients relapsed (15,19). Unfortunately, melarsoprol MICs for strains from the M'banza Congo focus have not yet been measured, but trypanosomes from this focus are likely intrinsically more resistant to melarsoprol than elsewhere.

These findings, although reassuring, underline the necessity of a strengthened surveillance system for African trypanosomiasis, through which secular trends in the frequency of treatment failures among patients with *T.b. gambiense* trypanosomiasis would be monitored in a small number of sentinel centers. The World Health Organization is developing such a system (25). Its measures will need to take into consideration modifications in the baseline characteristics of patients (most of which will be a result of changes in the intensity of case finding), changes in therapeutic regimens, and the completeness of follow-up by using Kaplan-Meier analyses.
